# Multiple mining impacts induce widespread changes in ecosystem dynamics in a boreal lake

**DOI:** 10.1038/s41598-017-11421-8

**Published:** 2017-09-05

**Authors:** Jaakko Johannes Leppänen, Jan Weckström, Atte Korhola

**Affiliations:** Environmental Change Research Unit (ECRU), Department of Environmental Sciences, University of Helsinki, P.O. Box 65, FIN-00014, Helsinki, Finland

## Abstract

In order to satisfy the needs of constant economic growth, the pressure to exploit natural resources has increased. Since accessible mineral resources are becoming scarce, the mining industry is constantly looking for novel techniques to allow commercial exploitation of lower-grade deposits. However, mining can have considerable impacts on freshwater ecosystems. Here, we present the ecological damage inflicted by mine water originating from the massive Terrafame Talvivaara polymetal mine (central Finland), where bioheap leaching is being applied to high-sulphur low-grade ore. We found that saline mine water has turned the lake meromictic, and sediment is heavily contaminated. As a result, important zooplankton and phytoplankton groups have been significantly altered. As the exploitation of poor-grade deposits is the future of the mining industry globally, water management should be taken to a higher level in order to proceed towards a sustainable mining sector.

## Introduction

The world is thirsty for key metals due to the high demand of technological societies. However, if new high-grade or large low-grade deposits are not found in the near future, a deficit of supply may be expected^1^. Despite its importance, the mining industry is acknowledged as being a high-risk activity in terms of environmental safety. Some of the worst contamination accidents during recent decades have been mining disasters, such as the dam failures of Aznalcollar, Spain, in 1998^2^, Baja Mare, Romania, in 2000^3^, and Bento Rodrigues, Brazil, in 2015^4^. Uncontrolled mine water discharges are thus acknowledged as a significant source of surface water pollution and therefore an issue of great concern^5^. The high magnitude of damage inflicted by recent mine water accidents is due, at least partly, to the technological advances of the past decades; it has become economically feasible to extract low-grade ores, which has resulted in larger mines and unprecedented high volumes of mine waste water. In fact, total mineral production has doubled in the past three decades^6^.

The Terrafame Talvivaara Ni-Zn-Co-Cu open pit mine, located in central Finland, is a warning example of pollution problems linked to modern mining, where low-grade deposits are being exploited. Bioleaching^7^ is a proven extraction method in low-grade Cu deposits, whereas Terrafame Talvivaara (established in 2007) is one of the first operational polymetal mines to use the technique of heap bioleaching. The heap/stockpile bioleaching is an extraction procedure, where metals are extracted from ore heaps by microorganims^7^. The volume of the Terrafame Talvivaara Ni sulphide ore deposit, in terms of total ore, has been reported to be the second largest in the world (1004 Mt). The planned annual production of 30 000 t of Ni^8^, with an average concentration of 0.26%^9^, is equivalent to approximately 11 Mt total ore. However, the ore (black schist) is of very low grade and the elemental Ni concentration (0.26%) is one of the lowest in operational sulphide Ni mines^10^. In contrast, the black schist of Terrafame Talvivaara is very rich in S (9.1% in metal-rich black schist^11^), and black schist is considered to be one of the most easily weathered rocks in Finland^12^. In addition, in order to speed up the extraction process, the Terrafame Talvivaara ore is treated via the additional input of sulphuric acid^13^, which further increases the amount of total S in the mine water. The mine water management of Terrafame Talvivaara is based on water recycling. According to the original plan, the remaining unusable waste water was to be purified chemically and channeled through a settling pond and wetland treatments prior to release into the environment^14^. The waste water treatment, however, has thus far not operated as originally planned.

**Figure 1:**
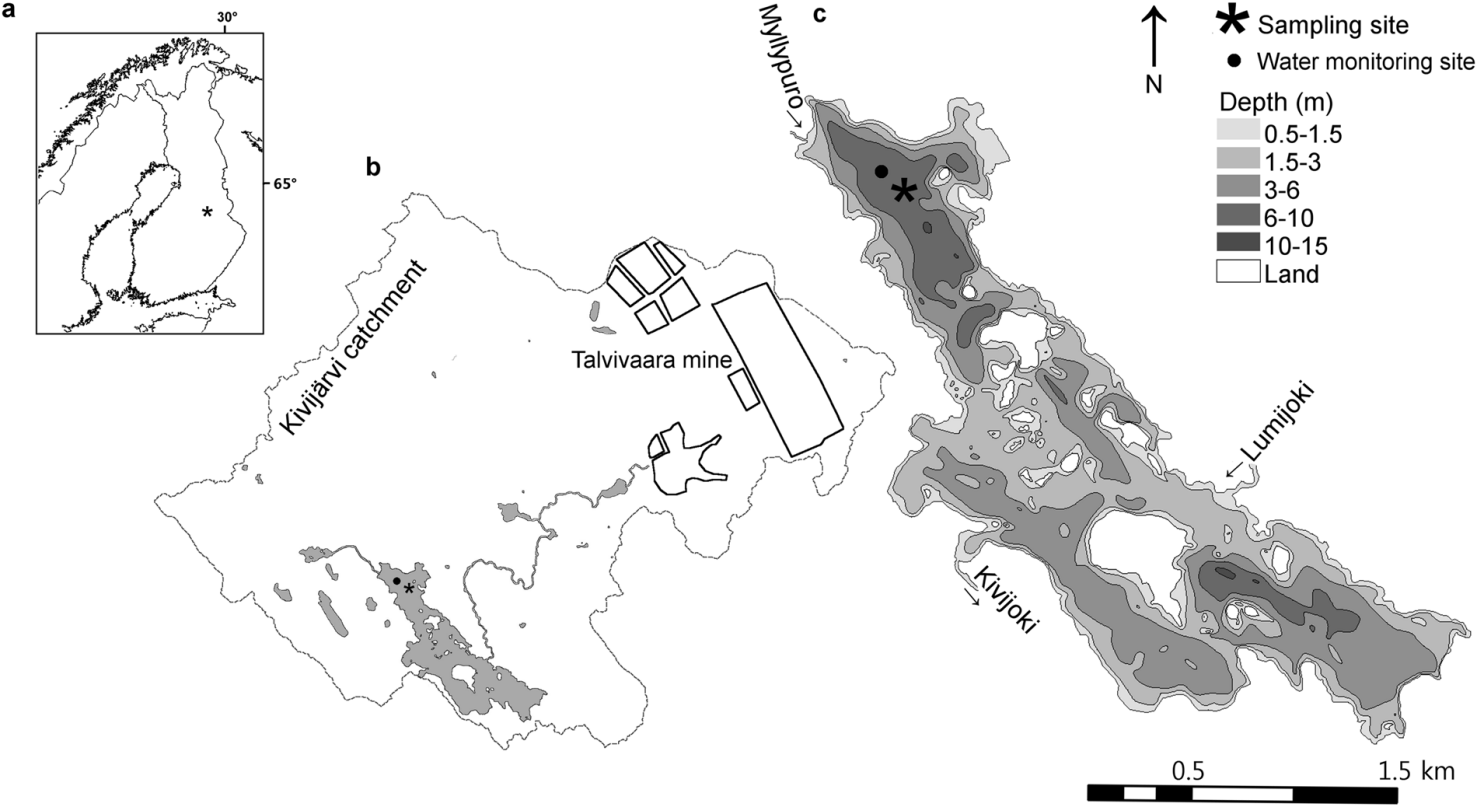
Study site location and lake characteristics. (**a**) Lake location in central Finland, (**b**) catchment boundaries, Terrafame Talvivaara mine and main channels, and (**c**) bathymetry of Lake Kivijärvi and location of data collection sites. The data for the map was retrieved from the National Land Survey of Finland open data databank (http://www.maanmittauslaitos.fi/en/e-services/open-data-file-download-service) under the open data CC 4.0 license (https://creativecommons.org/licenses/by/4.0/), and customized in ArcMap, Version 10.3.1 (http://desktop.arcgis.com/en/arcmap/) and in Corel Draw X8, version 18.0 (http://www.coreldraw.com/en/product/graphic-design-software/).

Lake Kivijärvi, located a few kilometers downstream from the mine (Fig. 1), has been affected by controlled mine water discharge and numerous unintentional leaks since 2007. Mining sewage water has consisted mainly of sulphate (SO^4^_2−_), Na and metals. During the second half of 2012, excess rainwater and raffinate (process waste) was stored in the gypsum pond, which suffered a catastrophic leak in November 2012. Some 240 000 m^3^ of saline and metal-containing (150 t of Fe, 150 t of Mn, 2 t of Ni, 1 t of Zn, 70 kg of U, 60 kg of Co and 2 kg of Cd) waste water was released into the environment. Since the discharge flowed north and south, accurate initial concentrations of elements entering the southern route, and eventually Lake Kivijärvi, are not known. Acidity was partly neutralized on site by emergency liming^15^. According to the report released by the Finnish Environment Institute, the spill inflicted direct negative effects on the biota in the receiving lakes^16^. Lake Kivijärvi is the largest and the most distant of the impacted lakes on the southern route. The 2012 accident was soon followed by another major leak in 2013. In 2016, the Terrafame Talvivaara mine obtained a permit to release waste water into a large lake north of the mine. According to the regional authorities, the deep parts of the lake are already suffering from meromixis-induced anoxia^17^.

Surprisingly, despite active and sometimes heated public discussion regarding the environmental and economic viability of the mine, and the fact that many other low-grade high-sulphidic deposits are going to be activated in the near future^1^, the environmental damage directly caused by Terrafame Talvivaara has not yet been fully assessed scientifically.

In this study, we combined limnological times-series data, land use history, and palaeolimnological data from Lake Kivijärvi to evaluate the ecological impact of the uncontrolled mine water discharges from the large-scale but low-grade open pit Terrafame Talvivaara mine on the lake. The major bioindicators used to track the traces of mining pollution were diatoms (algae) and Cladocera (crustacean zooplankton), both known to occupy important niches in aquatic systems and thus reflecting extremely sensitively the state of a waterbody. Both groups are also known to respond quickly to environmental changes and they have successfully been used previously in tracing mining pollution impacts^18, 19^. Additional palaeoecological information is derived from sediment geochemistry. The integration of limnological and palaeolimnological approaches is of prime importance for effective ecosystem management, as it allows both pre-disturbance conditions and the present ecological state of the lake to be assessed. This is the first attempt to address the impacts of a large-scale open pit mine on a freshwater ecosystem using the novel bioleaching extraction technique. It provides valuable background information for authorities regarding plans of opening new open pit mines with a similar framework.

## Results

### Limnological monitoring records

**Figure 2:**
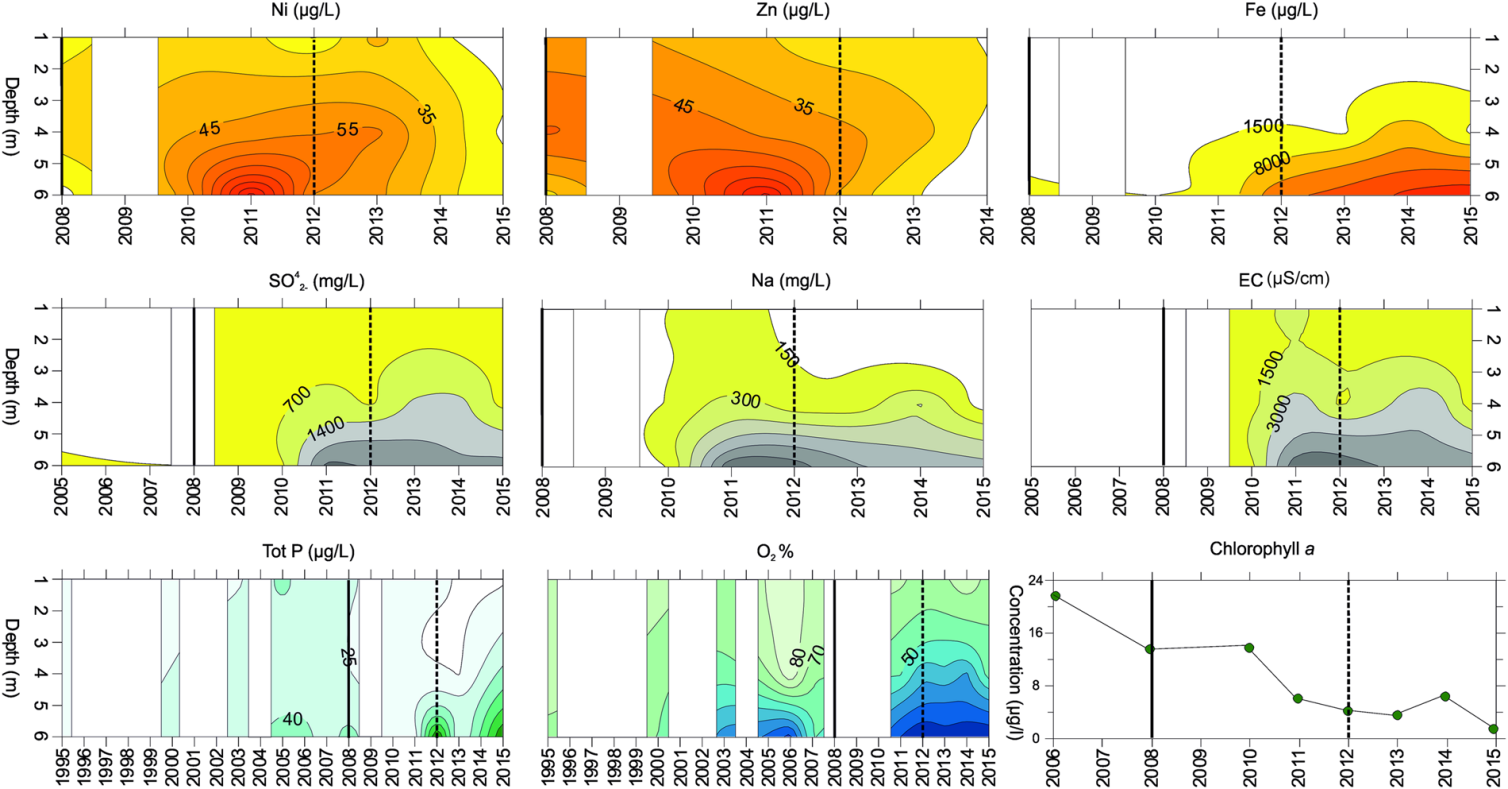
Lake Kivijärvi’s water chemistry characteristics. Variation of Ni, Zn, Fe, sulphate (SO^4^_2−_), Na, electrical conductivity (EC), total phosphorus (Tot P), oxygen (O_2_) and chlorophyll *a*. The y-axis indicates water sampling depth, except for chlorophyll *a*, where it indicates concentration. The solid vertical line indicates the beginning of mining activities, and the dashed vertical line indicates the 2012 gypsum pond accident. Detailed values and sampling dates, and concentration data regarding pH, Ca and total nitrogen (N) are available online (Supplementary Fig. 1). Data were retrieved from: http://www.syke.fi/avoindata. Data Source: Finnish Environment Institute and the Centres for Economic Development, Transport and the Environment (ELY Centres).

According to the limnological monitoring data (Fig. 2, Supplementary Fig. 1), Lake Kivijärvi has shifted from a mesotrophic state towards more oligotrophic conditions during the past decade (2005–2014), and it is characterized by clearly elevated hardness (as measured by electrical conductivity) and also by very high Ca concentrations (Supplementary Fig. 1). Saline mine water has created a clear chemocline (Supplementary video), which currently resides at a depth of ca 4 m, hindering water circulation between the consequently established monimo- and mixolimnion. Hypoxic or anoxic conditions have prevailed in the isolated deep monimolimnion since 2011. In addition, high concentrations of SO^4^_2−_ and Na in the deep-water layer confirm the development of a strong chemocline. This is further reflected as elevated and very high concentrations of P and Fe in the monimolimnion. The concentrations of Ni, Zn, Na and SO^4^_2−_ increased at all water depths during the early years (2008–2011) of mining activity. A temporary decrease in pH (Supplementary Fig. 1) below the chemocline is also clear, whereas pH in the mixolimnion has remained relatively stable. The impact of the gypsum pond accident (late 2012) can be seen as an increase in the concentration of Zn and Ni, with a simultaneous drop in pH at a water depth of 4 m (Supplementary Fig. 1). The concentration of Ca increased at the beginning of 2013 due to the gypsum pond leak and the emergency liming procedures (Supplementary Fig. 1). Ni and Zn concentrations decreased in the post-2014 water samples (Fig. 2). The water volume below the chemocline currently represents approximately 10% of the total lake water volume and covers approximately 13% of the total bottom area of the lake. According to monitoring reports^20–22^, the mine water has affected the lake biota in many ways. Since the start of mining, blooms of the freshwater alga *Gonyostomum semen* have occurred and the maximum summer phytoplankton biomass has declined. In addition, deformed small cryptophytes were detected in the plankton samples of the water column in 2013^20^. Moreover, since 2008, the benthic invertebrate community has changed drastically in the deep zones^21^. The effects of mine water on the higher trophic levels are currently unknown, but according to recent report^22^, fish in Lake Kivijärvi have not accumulated abnormal levels of heavy metals in their tissues. However, studies regarding other potential impacts on fish, such as those induced by reduced food availability^23, 24^, have not yet been conducted in Lake Kivijärvi.

### Sediment chronology and geochemistry

**Figure 3:**
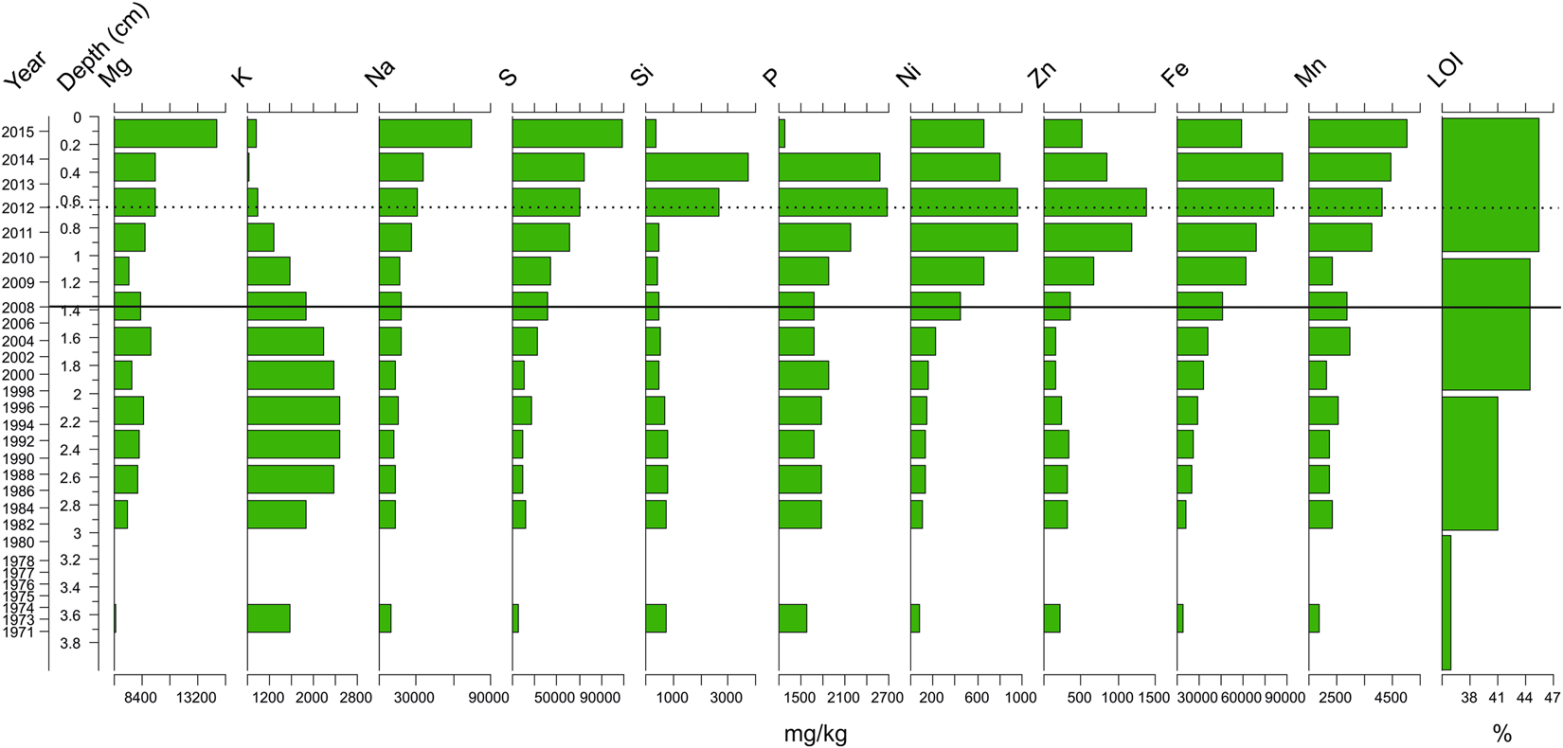
Lake Kivijärvi’s sediment geochemistry. The solid horizontal line indicates the beginning of mining, and the dashed horizontal line indicates the 2012 gypsum pond accident.

The highest values of unsupported ^210^Pb appear at a depth of 3 cm whereas distinct peak of ^137^Cs and traces of ^241^Am is located at 4.5–4.75 cm. Thus, the sediment chronology lacks reliability at the topmost samples. Below the sediment depth of 3 cm, the unsupported ^210^Pb concentrations decline nearly exponentially with depth suggesting relatively uniform accumulation of ~0.09 cm/yr (Supplementary Fig. 2). Several elements (Mg, Na, Mn and S) display elevated concentrations towards present, with highest concentrations at a depth of between 0 cm and 0.25 cm. Also P, Si, Fe, Ni and Zn are characterized by recent peaks. Mine derived S, Ni and Zn exhibit an increasing trend beginning at around 1.25–1.5 cm, which can be used to strengthen the age-depth model and to pinpoint the approximate depth of the beginning of the mining activities (2008). The Si peak near the surface also supports the dating of the top samples. K reaches its highest concentration at 2–2.5 cm. Loss-on-ignition (LOI) analysis exhibits a slight increase from 3 cm (ca 1980) towards present (Fig. 3).

### Changes in sedimentary cladoceran and diatom communities

**Figure 4:**
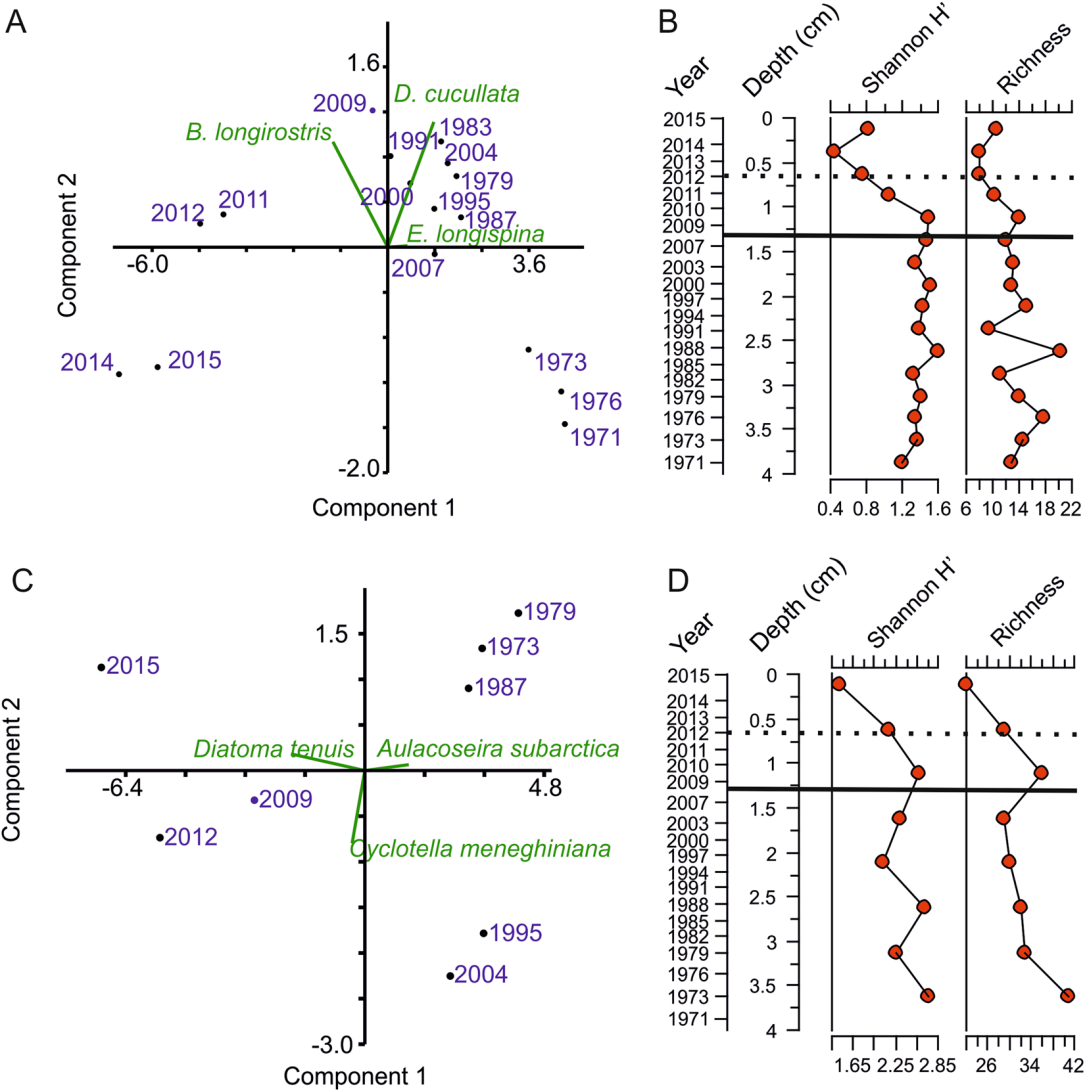
Changes in sedimentary cladoceran and diatom communities. (**A**) Cladoceran community data presented as a principal components analysis (PCA) biplot, (**B**) Chronology, rarefied species richness and Shannon H′ indices, (**C**) diatom community data presented as a PCA biplot, and (**D**) Chronology, rarefied species richness and Shannon H′ indices. The solid horizontal line in (**B**–**D**) indicates the beginning of the mining, and the dashed line indicates the 2012 gypsum pond accident.

**Figure 5:**
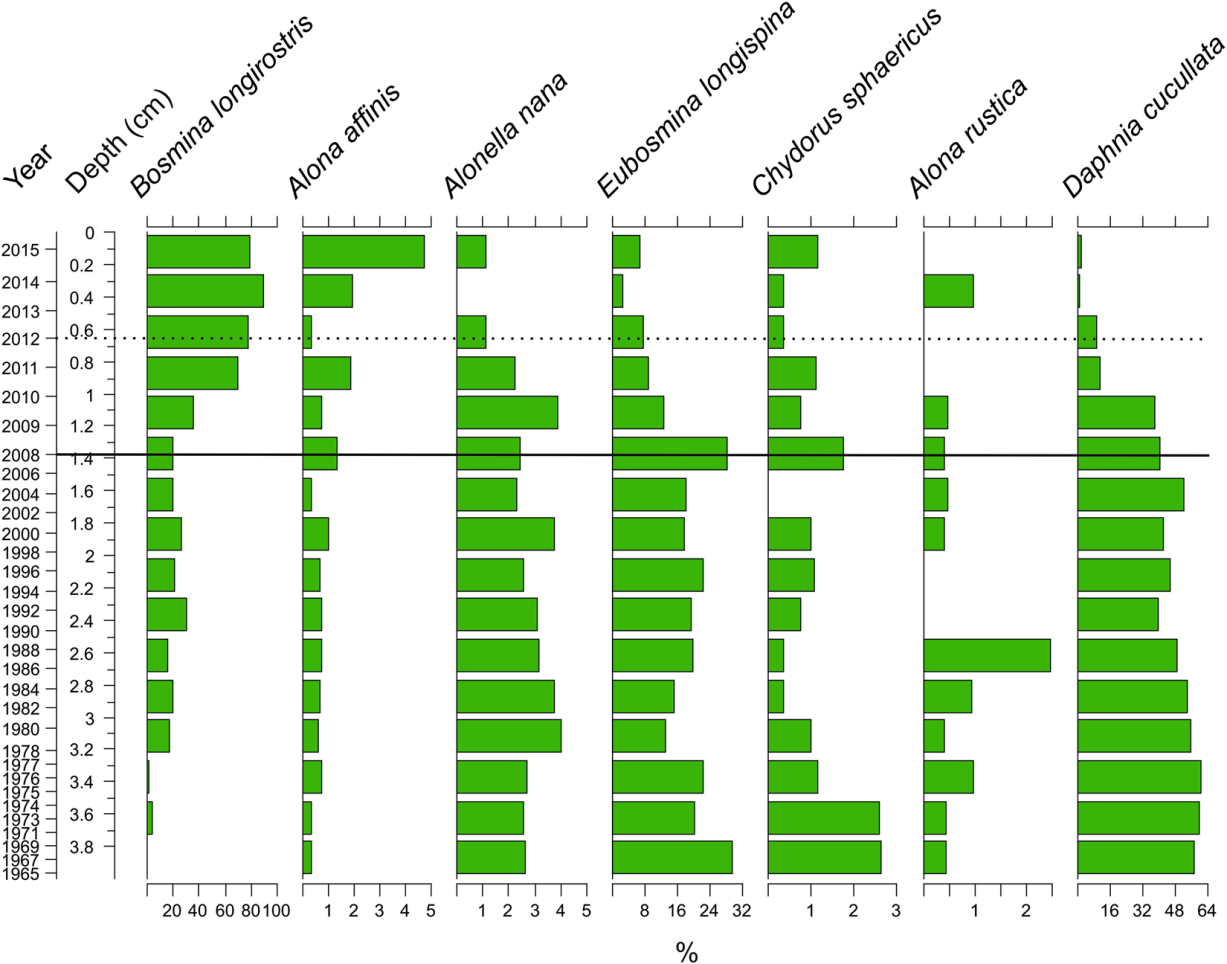
Cladoceran Stratigraphy. Cladoceran stratigraphy. Only the most abundant taxa with the proportional abundance of >2% is shown. The solid horizontal line indicates the beginning of the mining, and the dashed line indicates the 2012 gypsum pond accident.

**Figure 6:**
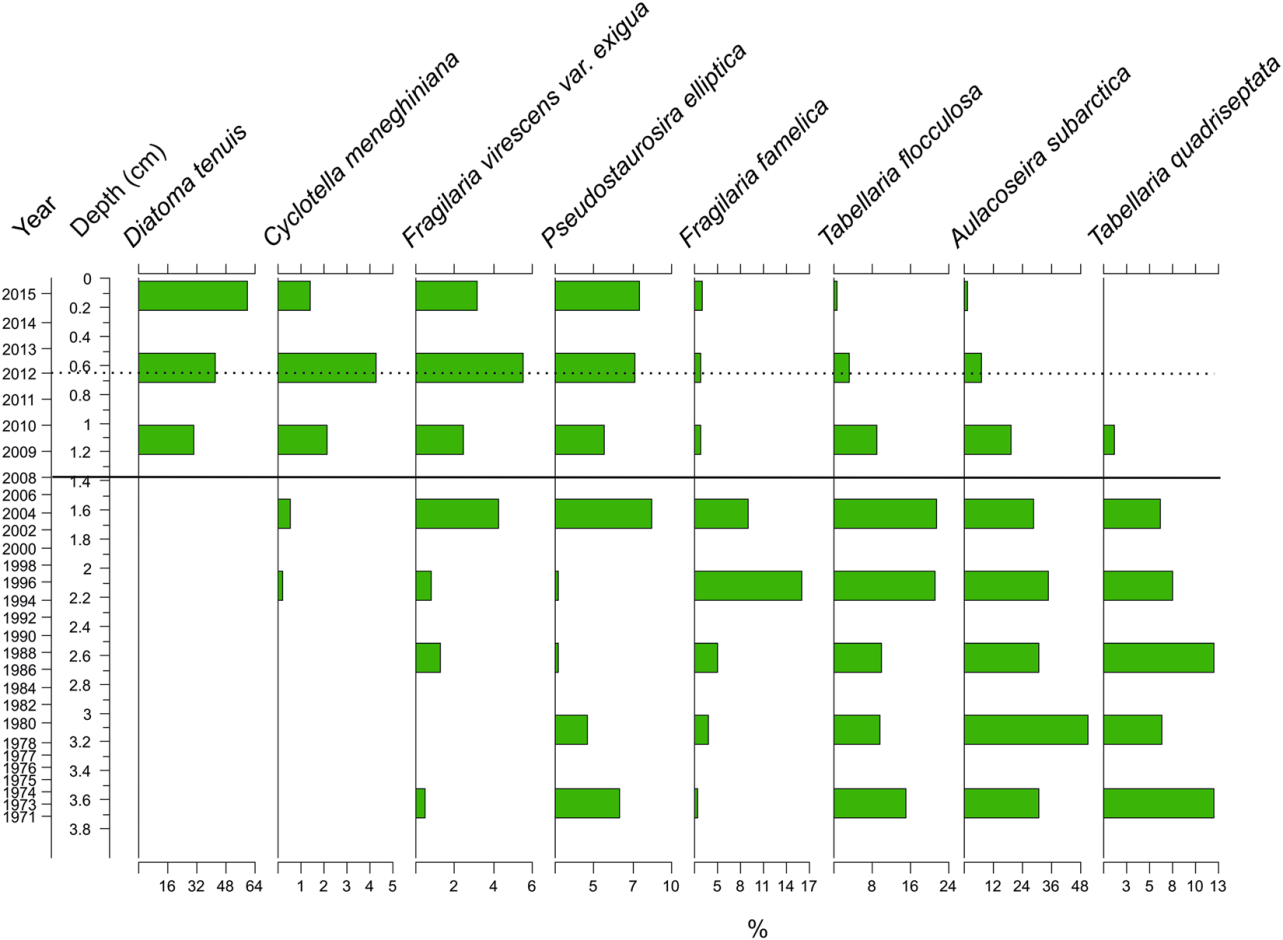
Diatom stratigraphy. Diatom stratigraphy. Only the most abundant taxa with the proportional abundance of >4% is shown.The solid horizontal line indicates the beginning of the mining, and the dashed line indicates the 2012 gypsum pond accident.

Lake Kivijärvi’s sedimentary cladoceran remains were numerous and exceptionally well preserved. Throughout the sediment core, the cladoceran community was dominated by pelagic species, namely *Daphnia cucullata, Eubosmina (Bosmina) longispina* and *Bosmina (Bosmina) longirostris*. The cladoceran community structure has been significantly altered by the mining discharge (analysis of similarities, ANOSIM, R = 0.8143, *p* = 0.0002) (Figs 4 and 5), and species richness and diversity have drastically decreased since the onset of mining activity – from 13.9 (standard deviation, SD, 2.9) and 1.40 (SD 0.10) to 10.2 (SD 2.4) and 0.92 (SD 039), respectively (Fig. 4B). A total of 113 diatom taxa belonging to 25 genera were detected in the sediment of the lake. Similarly to cladocerans, the diatom community structure has been significantly altered by the mining discharge (ANOSIM R = 0.9282, *p* = 0.0179) (Figs 4 and 6). Diatom species richness and diversity have decreased since mining activity began (2008) – from 33 (SD 4.7) and 2.4 (SD 0.27) to 29 (SD 7) and 2.0 (SD 0.59), respectively (Fig. 4D). In both groups, the post-mining samples plot to the left side of the principal components analysis (PCA) biplot, indicating pronounced changes in both communities (Fig. 4A,C).

## Discussion

We used limnological monitoring data and palaeorecords from Lake Kivijärvi to assess the longer-term dynamic pattern of the lake ecosystem and to identify the start of its degradation due to mining activities. The elemental concentrations in the sediment of the lake are in good agreement with known land use history of the lake catchment. The only clear change in sediment geochemistry before the mining activity is the elevated concentration of K during the 1990s, which is most probably a result of increased catchment erosion due to intense ditching activities between 1970 and1990^25, 26^ (Fig. 3). The low values of ^137^Cs and ^210^Pb in the topmost samples is probably caused by dilution by mine-derived minerals^27^. Therefore, the dates for the topmost samples are uncertain. However, because Ni and Zn concentrations are elevated already at the depth of 1.25–1.5 cm (Fig. 3), this section can be regarded to correspond to the beginning of the mining impact at 2008. We used the depth of 1.375 cm to correspond to the year (2008) and calculated the constant sedimentation rate of 0.19 cm/yr from the depth of 1.5 cm to the sediment surface. In addition, the peaks of Si in samples between 0.25–0.75 cm highlight nicely the late 2012 and 2013 mine accidents suggesting a relatively good age-depth model. The sediment ages below the depth of 1.5 cm are based on the age-depth model provided by the University of Liverpool’s Environmental Sciences Laboratory (Supplementary Fig. 2).

During the pre-mining period, both the cladoceran and diatom communities were relatively stable, representing the natural background conditions (Figs 4–6). The cladoceran assemblages were characterized by high species richness and diversity, and consisted of species common in European boreal fresh waters^28^. The same features can also be seen in the diatom assemblages, where a richer and more diverse diatom community occurred before the mining activities.

The widespread mining pollution impact is clearly visible in the water monitoring data, in the elemental concentrations of the sediment samples, and in the sedimentary biological data, indicating a damaged aquatic ecosystem. Sediment elemental concentration data (Fig. 3) support the water chemistry data as each sediment sample is comprised of months of accumulation thus minimizing the effects of short-term fluctuations in water quality. Even though many elements are mobile in sediments^29^, mining-related elemental concentration (Ni, Zn, Fe, S, Na) peaks in the most recent samples indicating the onset of pollution and the dam accidents. In addition, the very high concentrations of Si in the most recent samples, likely derived from the mining process^30^, provide a minerogenic fingerprint of the dam accidents in 2012 and 2013. A lower concentration of P in the topmost sediment sample probably reflects the anoxic conditions generated in the monimolimnion, which have resulted in a release of sedimentary P into the water column (Figs 2 and 3).

The beginning of the severe pollution is clearly visible in the water samples of 2010–2011 (Fig. 2) and, in particular, Ni and Zn concentrations exhibited distinct peaks at all sampling depths (Supplementary Fig. 1). Simultaneously, many parameters (e.g. electrical conductivity, SO and Na) show the beginning of meromixis and the deterioration of the hypolimnion due to increased concentrations of saline and dense mine water (Fig. 2). Mine water-induced meromixis has been reported earlier from a bay in Camp Lake, Canada, which became meromictic due to mine drainage originating from highly sulphidic tailings^31^. In Lake Kivijärvi, the strength and stability of the chemocline was distinctively demonstrated during the 2012 dam disaster as the burst of leaking mine water did not mix with the hypolimnion, but instead spatially dispersed around the lake in the mixolimnion (Fig. 2, Supplementary Fig. 1). The chemocline has efficiently hindered the circulation of nutrients between the bottom and upper water layers, resulting in clearly decreased chlorophyll *a* and P concentrations in the mixolimnion (Fig. 2). This shift in primary production may have had a negative effect on cladoceran food quality and quantity, hence reducing the species diversity as documented by the sedimentary data. A similar event has also been documented in Canada, where seepage of road salt resulted in a permanent halocline, which in turn hindered the nutrient cycling from the hypolimnion to the epilimnion, causing a shift from a nutrient rich system to an oligotrophic lake^32^.

The most striking feature in the cladoceran community was the rapid displacement of *D. cucullata* by *B. longirostris* (Fig. 5) at sediment depth of 1 cm, which corresponds to ca. 2010. Even though the meromixis alone cannot explain the collapse of the *Daphnia* population^33^, it may have had at least some negative impact, because *Daphnia* relies on vertical migration to avoid predators^34^. In addition, the shift in primary production may have had an impact on *Daphnia*. However, due to the lack of data regarding the food web structure in Lake Kivijärvi, it is difficult to postulate the role of possible changes in the top-down or bottom-up dynamics.

In Lake Kivijärvi, the most important pollutants are the highly toxic Ni and Zn^35^, which, in the prevailing pH^36^ are regarded as particularly harmful to the biota^37^. While the highest measured surface water Ni (53 µg/L) and Zn (68 µg/L) concentrations are below the reported toxicity limits for *Daphnia* in hard water environments, the mixture toxicity^38^, transgenerational effects^39^ and the dietary route^40^ may induce toxic effects in lower Ni and Zn concentrations. The highest SO^4^_2−_ concentration (1200 mg/L) in the mixolimnion of Lake Kivijärvi is also below the reported acute toxicity thresholds of *Daphnia* and *Ceriodaphnia* ^41^. However, again, in a chronic reproduction assay, *Ceriodaphnia dubia* exhibited decreased SO^4^_2−_ tolerance (IC_50_ 843 mg/L) in hard water (320 mg/L CaCO_3_) suggesting an increased stress due to elevated total salinity^42^. In addition, gypsum karst lakes with high concentrations of dissolved solids, Ca and phosphates are clearly inhospitable environments for cladocerans^43^. The reason for the depleted cladoceran community in those lakes is SO^4^_2−_ salts, which reduce filter feeding efficiency^44^. In cladoceran reproduction assays, the negative effects were detected in lower salinity values (measured as electrical conductivity) than those reported in Lake Kivijärvi surface water^45^. The declining *D. cucullata* population or the drop in community indices is most probably a result of multiple stressors. Also, the extremely high water hardness in Lake Kivijärvi complicates the comparison between the results of this study and toxicity studies conducted in laboratories. Even though metal concentrations have slightly decreased in the most recent samples, the water quality will likely remain poor for a prolonged period. The recovery of the cladoceran community from sedimentary egg banks will also be hindered due to high toxicity of sediments^46^.

The impact of the mine can be first seen in the diatom samples at the depths between 1–1.25 cm, which corresponds most likely to 2009–2010 (Figs 4 and 6) as diatoms are primary producers and, in general, react faster to environmental changes than invertebrates^47^. The impact of the Terrafame Talvivaara mine effluent was clearly visible among some species, such as *Diatoma tenuis* (relative abundance up to 70%) and *Cyclotella meneghiniana*, which appeared suddenly and other taxa previously dominating the lake diatom community decreased (Figs 4 and 6). Both of these new dominants are known to thrive in slightly saline, high conductivity waters^48^. Although diatoms have been used widely in metal contamination studies due to their sensitivity to abrupt environmental changes^49–51^, most of these studies have been conducted in heavily contaminated ecosystems. It has been demonstrated that in sites with lower contamination, other perturbations (e.g. eutrophication) can override the signal of the effects of the metal/s^52, 53^. However, within the post-mining period, the most significant changes in the diatom community were caused by the mine-derived salinity increase rather than the increase in toxic metal concentrations *per se*. Differences in the sensitivity of organisms to elevated salinity may partly explain the recent shifts among cladocerans but particularly in diatom species assemblages, as salinity is regarded as one of the strongest environmental variables controlling the distribution of diatoms^54^.

Our results imply that salinity most likely also had an indirect impact on biological communities as meromixis inevitably decreased the oxygen and light levels at the lake bottom. This may have switched the diatom community structure from benthic to pelagic by decreasing favourable habitats for the benthic diatoms and thus enabling the dominance of planktic species such as *D. tenuis* (both epiphytic and in plankton^55^) and *C. meneghiniana*.

The distinct ecological changes in Lake Kivijärvi are a warning example of the risks associated with modern mining, where water/waste water volumes both in extraction processes and during spring time meltwater and storm water episodes are usually very high. The multiple impacts resulting from salinity and metals have shifted Lake Kivijärvi from a healthy boreal lake and to a badly degraded lake. The health of the water basin has suffered similarly. In the near future, the bioleaching technique may potentially be applied to black shale and schist deposits in many countries (Sweden, Poland, the Czech Republic, France, Canada, the USA, Argentina, India, Pakistan, China, South Korea, Australia, South Africa and Zambia)^1^. Our results suggest that mining companies should pay special attention to the management of saline mine waters when large low-grade sulphidic deposits are going to be mined, especially in areas where precipitation exceeds evaporation.

In conclusion, we have shown that since 2008, the pollution derived from the Terrafame Talvivaara mine has had a pronounced impact on the aquatic ecosystem of Lake Kivijärvi (Table 1). The direct effects of waterborne heavy metal pollution are probably mitigated due to elevated hardness, but other, less straightforward processes, such as the dietary route of toxicants and over-generation effects may have had a greater impact on the lake biota. Another important damaging factor includes osmotic stress due to increased salinity. In Lake Kivijärvi, where a mixture of many potentially harmful substances is simultaneously introduced into the aquatic ecosystem, it is difficult to pinpoint any single factor behind the massive ecological disruption. However, in addition to the toxic impacts of pollution, the mine water-induced meromixis may have played a critical role in the food web changes. In addition, the hypolimnion was cut off from the productive surface layer resulting in a vicious circle of starvation throughout the food web. Our findings strongly imply that the saline non-acidic mine water can be regarded as dangerous as classic acidic mine waters, even in cases where direct water toxicity is at least partly attenuated by the protective role of the constituents of the mine water itself. In many countries, environmental regulations limit the heavy metal load in mine water to supposedly non-harmful levels. However, in the modern mining industry, water volume also becomes an issue because gigantic mines have to handle high amounts of water. If water management is not sufficiently handled in large sulphide mineral mines, disasters such as the one in Talvivaara will overshadow aquatic ecosystems all around the world.

**Table 1:** The main time periods of Lake Kirkkojärvi ecosystem.

Period	Years	Water characteristics	Sediment chemistry	Cladocera	Diatoms	Interpretation
After the accident	2013–2015	Lake is meromictic. High salinity prevails. Fe and P are released from the sediment to hypolimnion.	Fe and P exhibit lower concentrations, also Ni and Zn decrease due to decreased pollution.	*B. longirostris* dominates.	*D. tenuis* dominates	Lake is clearly impacted by pollution
Pond accidents	2012–2013	Strong stratification. Dam accident derived Ni and Zn stay on top of chemocline.	Ni and Zn peak. Si peaks due to the pollution accident.	*B. longirostris* dominates. Richness and diversity are low.	*D. tenuis* increases	Lake is clearly impacted by pollution
Mining begins	2008–2012	Onset of meromixis and anoxia in deep water. Mine-related elements increase.	Zn, Ni, S and Na increase	*Bosmina longirostris* increases, diversity and richness decreases	*Diatoma tenuis* increases	Saline water and meromixis affects the system
Natural	1971–2008	Not permanently stratified, chemistry resembles natural conditions	K increases due to forestry	*Daphnia* dominates. Rich and diverse community	*Tabellaria* and *Aulacoseira* spp. dominate. Rich and diverse community	Most likely nearly natural boreal lake

## Materials and Methods

### Study area

Lake Kivijärvi (63°55.6′N, 27°54.3′E WGS84) is located at 165 m above sea level in central Finland. The lake has an area of 1.8 km^2^ with a maximum depth of 12 m and average depth of ca 5 m. Lake Kivijärvi is fed by River Lumijoki in the south and the Myllypuro brook in the north and it drains via River Kivijoki to Lake Laakajärvi. Waste water originating from the Terrafame Talvivaara mine flows via River Lumijoki through the small Lake Ylä-Lumijärvi and the Rötylampi pond and drains into the south-eastern part of Lake Kivijärvi. The length of the water route from the mine to Lake Kivijärvi is ca 7 km (Fig. 1). The bedrock of the catchment area (53 km^2^) mainly consists of granite gneiss, whereas younger granite dominates the western part of the catchment area^56^. The vegetation of the catchment area is dominated by coniferous forest and peatland. The human population in the Lake Kivijärvi catchment area is less than 20 inhabitants and the distance to the nearest town with a notable population size (Kajaani, population 30,000) is 30 km. The annual average air temperature in the region is 2 °C and the yearly rainfall approximately 600 mm^57^. The Lake Kivijärvi catchment area was retrieved from the Finnish Environment Institute’s online catchment area tool (http://paikkatieto.ymparisto.fi/value) and the bathymetric data were acquired from the National Land Survey of Finland open data databank (http://www.maanmittauslaitos.fi/en/e-services/open-data-file-download-service). Spatial calculations were performed using the software ESRI ArcMap 10.3.1.

### Water chemistry

We used a data set consisting of 12 aquatic variables (Ca, Na, SO, electrical conductivity, Ni, Zn, Fe, pH, P, N, chlorophyll *a* and oxygen) measured from three different water depths (0–1 m, 3–4 m and 6–8 m, except chlorophyll *a*, which was measured only from surface water) to examine the effects of mine water discharge on the water quality. Water sampling was conducted in varying intervals (January 1993, 2000, 2013; February 2003, 2007, 2012; March 2008, 2010, 2011, 2013, 2014, 2015; April 2012, 2013; May 2012, 2013; June 2011, 2012, 2013, 2014, 2015; July 2008, 2010, 2011, 2012; August 2006, 2008, 2010, 2011, 2012, 2013, 2014; September 2011, 2012, 2014; October 2005, 2011, 2012, 2013; November none; December 2012). The data were retrieved from the Finnish Environment Institute’s online databank: http://www.syke.fi/avoindata. The water chemistry data are based on the national lake monitoring program and the Terrafame Talvivaara monitoring programme and are measured from a single monitoring station, located approximately 200 m north-west of our sediment sampling site (Fig. 1). Sampling and analysis were conducted using standard methods in accordance with national guidelines^58^. The isopleth contours in Fig. 2 were produced using the Kriging method in Surfer 11 software and are based on 43 single measurements for each parameter, except for chlorophyll *a*, which is based on 8 measurements.

### Coring and subsampling

Four short sediment cores (A, B, C and D) were retrieved with a HTH-Kayak gravity corer^59^ and one core (core E) with a Limnos gravity corer from the northern part of the lake at a water depth of 9.1 m in March 2015. The total length of each core was approximately 25 cm. All HTH-Kayak cores were subsampled at 0.25 cm intervals for the first 4 cm, which was estimated to include the time of the 2012 gypsum pond incident. From 5 cm onwards, the cores were subsampled at 1 cm intervals. Core A was used for cladoceran, diatom, and dating analysis. Core B was combined with cores C and D in order to gain enough material for geochemical analysis in 0–1 cm samples. From 1 cm onwards core B was used for geochemical analysis. All HTH-Kayak cores were retrieved undisturbed and were combined visually. In all cores, the top 1 cm consisted of black sulphide gyttja overlain by a thin (1 mm) crust of dark grey precipitate. Below the depth of 1 cm, the sediment colour was light brown. Core E was subsampled at 1 cm intervals and used for LOI analysis. All cores were subsampled in the field and stored in plastic ziplock bags in a dark cold room at +4 °C within 12 hours of retrieval.

### Dating and geochemical analysis

Freeze-dried sediment samples from core A were radiometrically dated by the University of Liverpool’s Environmental Sciences Laboratory. The radiometric dating chronologies were calculated using the constant rate of supply (CRS) and the constant initial concentration (CIC) ^210^Pb dating models^60^ with additional analysis of ^226^Ra, ^137^Cs and ^241^Am^61^. LOI was measured at 1 cm intervals (core E) to assess the changes in water content and organic content following widely used methods^62^. Acid soluble concentrations of P, Si, Fe, Mn, Zn, Ni, K, Mg, Na and S were measured using inductively coupled plasma optical emission spectrometry (ICP-OES) after HNO_3_ digestion in accordance with EPA3051 and SFS-EN ISO 11885:2009 standards at Metropolilab Helsinki environmental laboratory, which is an accredited testing laboratory (FINAS T058).

### Cladoceran and diatom analysis

Cladoceran analysis was based on published procedures^63, 64^. Permanent microscopic slides were prepared from which the cladoceran remains were identified and counted under a light microscope with × 100–400 magnification. A minimum of 249 individuals was counted from each sediment horizon to investigate species composition.

Diatoms were prepared using H_2_O_2_ digestion and HCl treatment, and cleaned diatoms were mounted in Naphrax. A minimum of 300 diatom valves from each sample were identified and counted along random transects at × 1000 magnification. Diatom identification was based on comprehensive literature^48, 55, 65, 66^. More details concerning the procedure and taxonomic literature used is available^67^.

### Numerical analysis

The Shannon diversity index (H′) was calculated to assess the species diversity in samples. Species richness was calculated using the rarefaction procedure in order to standardize the number of counted taxa. PCA was used to interpret and summarize the major patterns of variation within species data. PCA was performed on square-root transformed species data using the program CANOCO 5.01^68^. The ANOSIM procedure, with square-root transformed species data, was used in order to test the statistical difference of cladoceran and diatom communities between the periods prior to and after the establishment (2008) of the Terrafame Talvivaara mine. The Bray–Curtis dissimilarity index was used in ANOSIM. Shannon H′ diversity, species richness and ANOSIM were performed using the program PAST 3.1^69^.

### Data availability

The datasets generated during and/or analyzed during the current study are available from the corresponding author on reasonable request.

## Electronic supplementary material

Supplementary information

Supplementary video
