# Supplementary material for: Multiple mining impacts induce widespread changes in ecosystem dynamics in a boreal lake
Source: Sci Rep. 2017 Sep 5;7:10581. doi: 10.1038/s41598-017-11421-8 (PMC5585241; doi:10.1038/s41598-017-11421-8)
Supplement: Supplementary file 1 — Supplementary information [file 41598_2017_11421_MOESM1_ESM.pdf]

**TITLE:**

# Multiple mining impacts induce widespread changes in ecosystem dynamics in a boreal lake

**Authors:**

**Jaakko Johannes Leppänen, Jan Weckström & Atte Korhola**

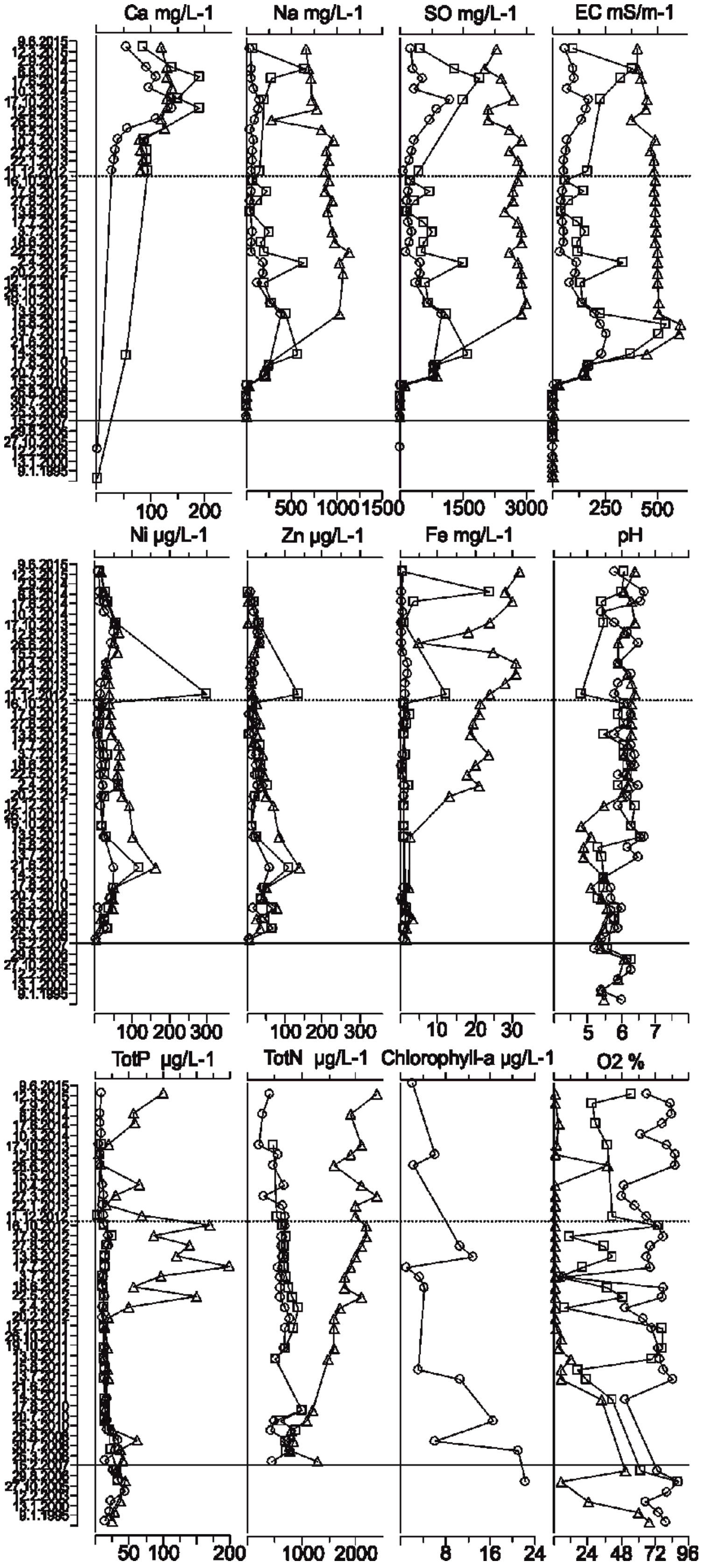


Supplementary Fig. 1. Lake Kivijärvi water parameters. Dates on Y axis are sampling dates. Circles represent 0-1 m surface concentrations, quadrats represent 3-4 m midwater concentrations and triangles represent 6-8 m deep water concentrations. Data retrieved from: http://www.syke.fi/avoindata. Solid line represents the beginning of the mining activity and dashed line represents 2012 dam accident.

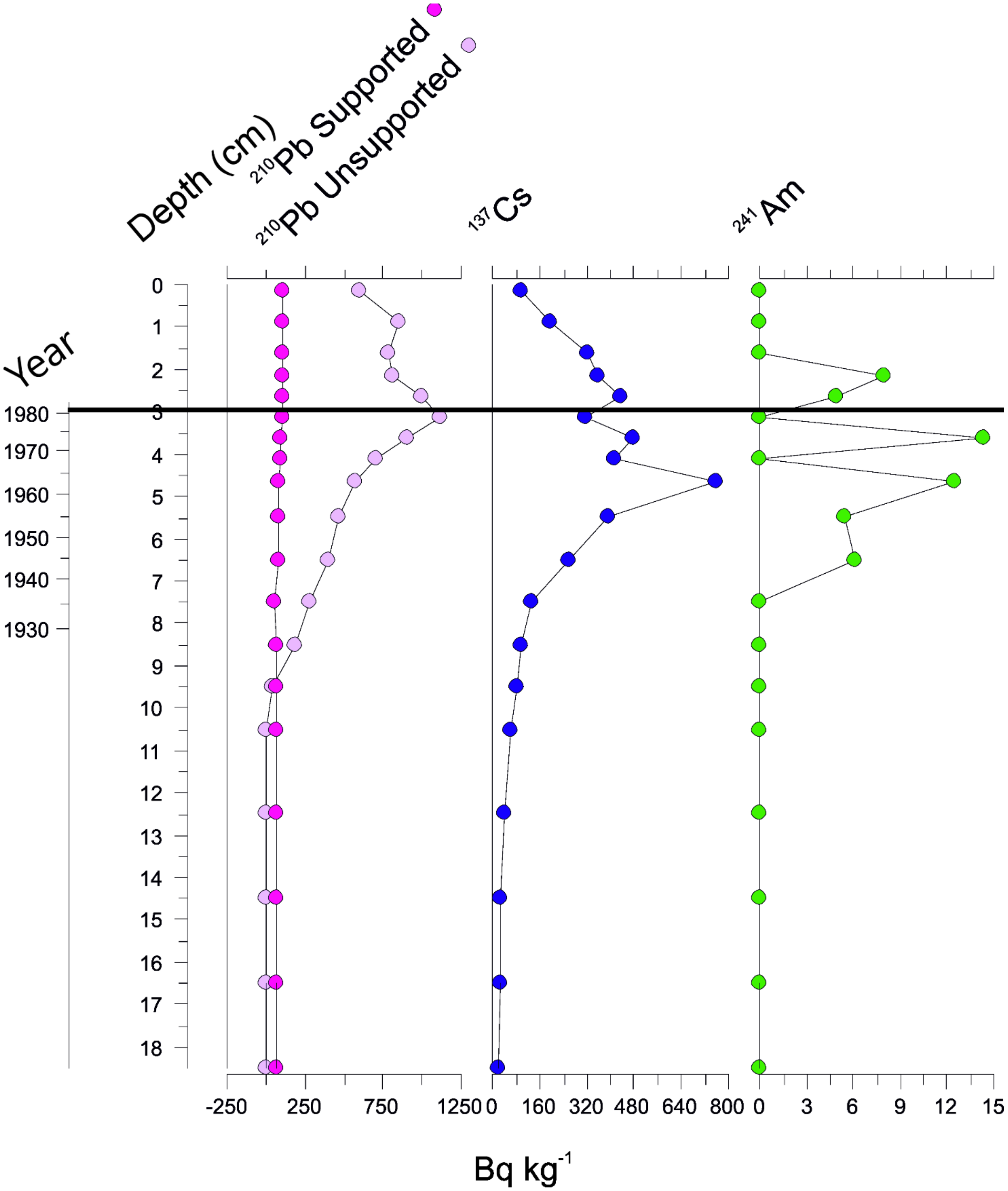


Supplementary Figure 2. Concentrations of Cs, Pb and Am in Kivijärvi sediment core and reliable dates. The horizontal solid line at 3 cm level denotes the limit of reliable dating. The age-depth model was conducted by Liverpool University.
